# Proton-Irradiation Effects and Reliability on GaN-Based MIS-HEMTs

**DOI:** 10.3390/mi15091091

**Published:** 2024-08-29

**Authors:** Zixin Zhen, Chun Feng, Hongling Xiao, Lijuan Jiang, Wei Li

**Affiliations:** 1 China Aerospace Science & Industry Corp Defense Technology R&T Center, Beijing 1000854, China; 2 Laboratory of Solid State Optoelectronics Information Technology, Institute of Semiconductors, Chinese Academy of Sciences, Beijing 100083, China; cfeng@semi.ac.cn (C.F.); hlxiao@semi.ac.cn (H.X.); ljjiang@semi.ac.cn (L.J.); wli@semi.ac.cn (W.L.)

**Keywords:** irradiation effects and reliability, GaN, MIS-HEMTs

## Abstract

A comprehensive study of proton irradiation reliability on a bilayer dielectrics SiN_x_/Al_2_O_3_ MIS-HEMT, the common Schottky gate HEMT, and a single dielectric layer MIS-HEMT with SiN_x_ and with Al_2_O_3_ for comparison is conducted in this paper. Combining the higher displacement threshold energy of Al_2_O_3_ with the better surface passivation of the SiN_x_ layer, the bilayer dielectrics MIS-HEMT presents much smaller degradation of structural materials and of device electrical performance after proton irradiation. Firstly, the least of the defects caused by irradiation suggesting the smallest structural material degradation is observed in the bilayer dielectrics MIS-HEMT through simulations. Then, DC and RF electrical performance of four kinds of devices before and after proton irradiation are studied through simulation and experiments. The smallest threshold voltage degradation rate, the smallest maximum on-current degradation and Gm degradation, the largest cut-off frequency, and the lowest cut-off frequency degradation are found in the bilayer dielectrics MIS-HEMT among four kinds of devices. The degradation results of both structural materials and electrical performance reveal that the bilayer dielectrics MIS-HEMT performs best after irradiation and had better radiation resilience.

## 1. Introduction

Gallium nitride-based high electron mobility transistors (HEMTs) have great potential in high-frequency, high-power, and microwave applications thanks to its wide bandgap, high breakdown electric field, high carrier density, and high carrier mobility at the hetero-interface [1,2,3,4,5]. However, the traps at the surface decrease the 2-dimensional electron gas (2DEG) density and lead to frequency characteristics degradation [6,7]. Moreover, the gate leakage is another problem limiting the current driving of GaN-based HEMTs. GaN-based metal–insulator–semiconductor (MIS)-HEMTs have been proposed to reduce traps and leakage [8].

For devices in space applications, more attention needs to be paid to the radiation effect, which will deteriorate the DC and RF performance of the device. Devices working in space are exposed to both particle and electromagnetic radiation, such as electrons, protons, neutrons, gamma rays, and alpha particles [9,10]. Irradiation can cause ionization radiation effects and displacement damage, which has been proven to be more severe in previous studies on GaN-based devices [11]. Therefore, we mainly concentrate on understanding and characterizing the displacement damage caused by proton radiation [10]. Typically, GaN HEMTs show the shift in threshold voltage; the degradation of cut-off frequency, saturation drain current, and extrinsic trans-conductance; and the increase in gate leakage after proton irradiation [12,13,14]. The magnitude of the degradation depends on the proton energy and fluence [12]. In recent years, research groups have conducted some research on proton irradiation effects on the common Schottky gate HEMT structure and single dielectric MIS-HEMT structure [15,16,17,18]. The bilayer dielectrics MIS-HEMT has emerged as a potential candidate taking advantage of the benefits of each layer in recent years. Comparative study on characteristics of AlGaN/GaN MIS HEMTs has been reported without proton irradiation [19] and with a certain fluence of proton irradiation [15]. However, the degradation of structural materials and frequency characteristics after proton irradiation are not reported in research about MIS-HEMT using the same bilayer structure [15,19]. Moreover, the influence of different proton fluence on GaN HEMTs needs to be explored. Therefore, it is necessary to study the proton irradiation effects on a bilayer dielectrics MIS-HEMT comprehensively.

Focusing on the problems mentioned above, four different proton fluences are adopted for a bilayer dielectrics MIS-HEMT and a single-dielectric-layer MIS-HEMT with SiN_x_ and with Al_2_O_3_, respectively, and the common Schottky gate HEMT for comparison in this paper. The degradation of structural materials and the degradation of device electrical performance are both explored through simulation and experiments. The results reveal that the bilayer dielectrics MIS-HEMT performs best after all four different irradiation fluences.

## 2. Device Structures

**Figure 1:**
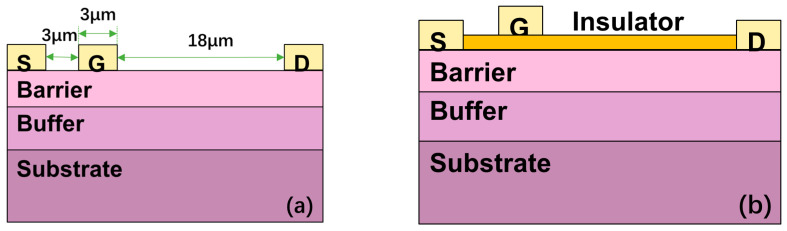
Cross-sectional schematic of (**a**) the Schottky gate HEMT and (**b**) MIS-HEMT.

Figure 1 shows the cross-section of the Schottky gate HEMT and MIS-HEMTs. Except for the dielectric layer, the Schottky gate HEMT and MIS-HEMTs are in the same structure. The device structure was grown on a sapphire substrate by a metal–organic chemical vapor deposition [19]. The epitaxial structure consists of a 25 nm AlGaN barrier layer and a 1.6 μm GaN buffer layer [19]. For the single dielectric MIS-HEMT, the insulator used in devices is SiN_x_ (10 nm) and Al_2_O_3_ (5 nm) separately [19]. For the bilayer dielectrics MIS-HEMs, a 10 nm SiN_x_ is deposited first, followed by a 5 nm Al_2_O_3_ layer. The SiN_x_ and Al_2_O_3_ layers are deposited by plasma-enhanced chemical vapor deposition (PECVD) and by atomic layer deposition (ALD) separately. The gate electrode is Ni/Au metals. The ohmic contacts (Ti/Al/Ni/Au) were formed through a rapid thermal annealing at 850 °C. The Lg, Lgs, and Lgd of the devices are 3 μm, 3 μm, and 18 μm, respectively [19].

The 2DEG densities and equivalent mobility(@Vgs = 0 V) of four fabricated devices are listed in the Table 1. The contact resistance is 0.54 Ωmm, which is measured through TLM.

Particle incidents may damage the material lattice, resulting in displacements, which will cause vacancies as well as other defects and influence device characteristics, so the degradation of structural material after proton irradiation was studied to start.

**Figure 2:**
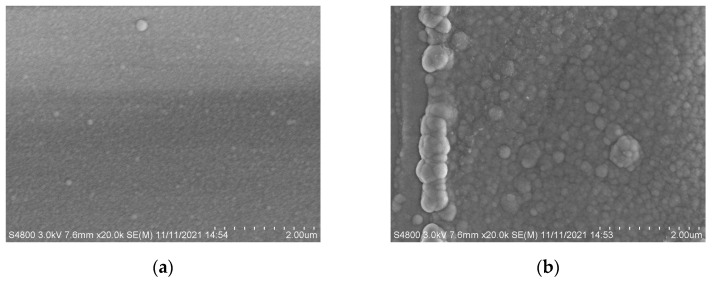
Experimental results of (**a**) surface morphology before irradiation and (**b**) surface morphology after irradiation.

To explore the degradation of structural material, firstly, we observed the surface morphology of the device structural material before and after proton irradiation, and found that surface morphology became rougher and more particles appeared, as shown in Figure 2.

**Figure 3:**
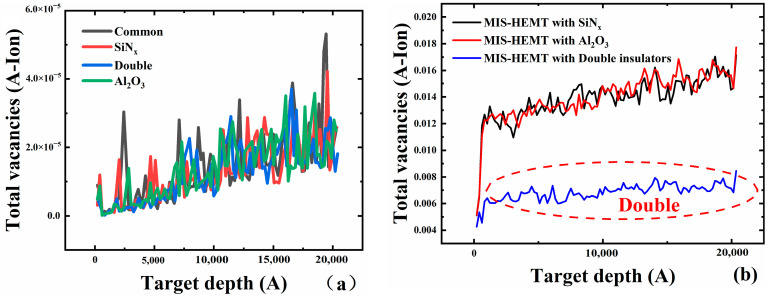
Comparison of defects (simulation) generated after proton irradiation of the common Schottky gate HEMT, single-dielectric layer MIS-HEMTs and bilayer layer MIS-HEMT: (**a**) low energy and (**b**) high energy.

Then, we calculated total dislocations, vacancies, and collisions in different structural materials through Stopping and Range of Ions in Matter (SRIM) simulation. Low-energy proton irradiation (3 MeV) was injected into the structural materials of the four different devices, and more total vacancies could be found in the common Schottky gate HEMT, as shown in Figure 3a. Fewer defects in MIS-HEMTs were displayed than in the common Schottky gate HEMT. To further explore the vacancies in the other three kinds of devices, proton irradiation with higher energy (10 MeV) was adopted. The total replacement, total vacancy, and replacement collision were calculated, and the results are exhibited in Figure 3b and Table 2. The least of the three defects was displayed in the bilayer dielectrics MIS-HEMT according to the simulation results, which suggests that the bilayer dielectrics MIS-HEMT can reduce structural material degradation.

**Figure 4:**
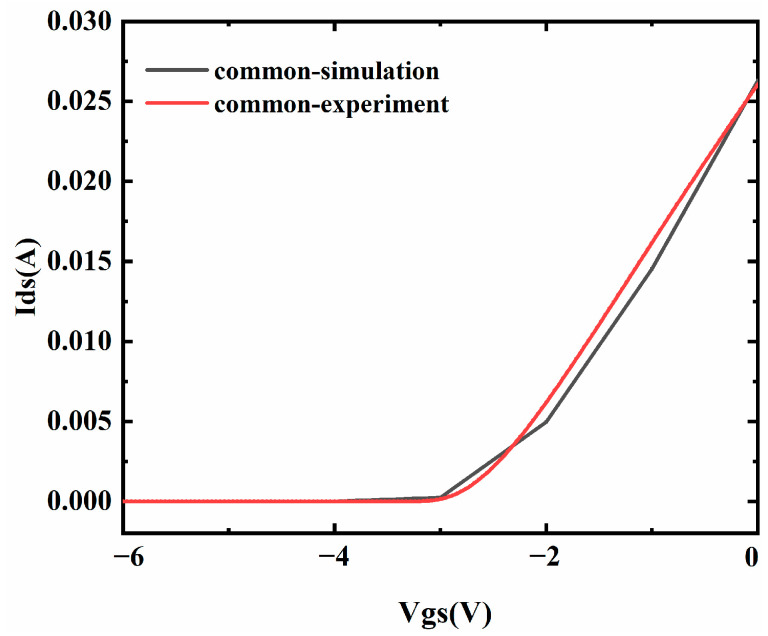
The transfer curves of the simulation and experimental results.

Two-dimensional device simulation was used to explore the electrical performance of four kinds of devices. Polarization models, the Shockley–Read–Hall model, the Auger model, and the concentration-independent mobility model were used in the simulation [20]. The devices structures are the same as that described in Figure 1. The simulation results fit well with the experimental results [15], as shown in Figure 4. 

## 3. Results and Discussion

On-current degradation in different structures after the 3 MeV proton irradiation fluence of 10^14^ is listed in Table 3. The maximum on-current degradation in the bilayer MIS-HEMT degraded the least, while the maximum on-current in the common HEMT showed the largest degradation. This may be related to the degradation of the carrier density and mobility. The degradation of the carrier density and mobility of the bilayer MIS-HEMT was 1.04% and 1.05%, while the degradation of the carrier density and mobility of the common HEMT was 14.94% and 45.43%.

The threshold voltages of four devices were studied, and the threshold voltage shift (△Vth) of different devices before and after different fluences of proton irradiation were compared in our simulations. The results of degradation rate of Vth (△Vth/Vth_0_) are shown in the Table 4. The degradation rate of threshold voltage in four devices increased as the proton irradiation fluence grew, which may be attributed to the defects induced by proton irradiation. The smallest degradation rates were found in the bilayer dielectrics MIS-HEMT regardless of proton irradiation fluence. Moreover, the change in degradation rate of Vth in the bilayer dielectrics MIS-HEMT was also smaller than that in the common Schottky gate HEMT. The degradation rate of the common Schottky gate HEMT grew from 15.1%@10^11^/cm^−2^ to 50.3%@10^14^/cm^−2^, while that in the bilayer dielectrics MIS-HEMT showed the smallest degradation rate and degradation rate change, from 10.6%@10^11^/cm^−2^ to 37.2%@10^14^/cm^−2^. Compared with the common Schottky gate HEMT, all three MIS-HEMT devices showed lower degradation rates of Vth. Since the active region in the common Schottky gate HEMT structure is closer to the surface, it is more vulnerable than in the MIS-HEMTs, so the degradation of the common Schottky gate HEMT structure is more serious. Among the three MIS-HEMT devices, the improvement in the threshold voltage shift of the bilayer dielectrics MIS-HEMT device was the most obvious, which strongly proves the effectiveness of the bilayer dielectrics layers. The higher displacement threshold energy of the upper Al_2_O_3_ decreased defects induced by particle incidence, and the lower SiN_x_ layer provided better surface passivation, reducing the generation of the secondary defects.

**Figure 5:**
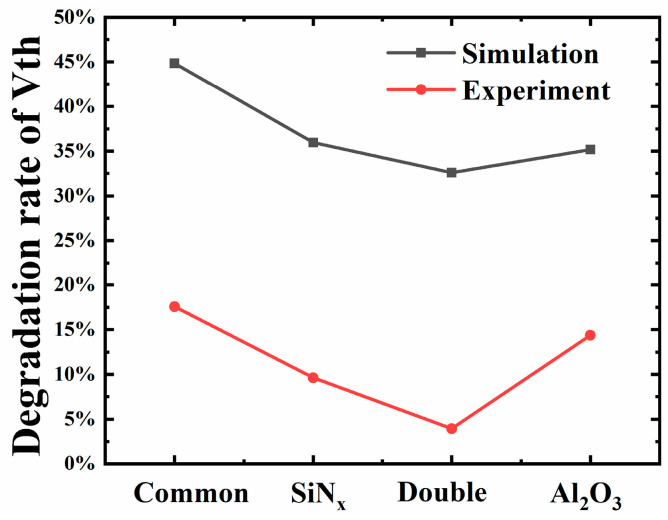
Comparison of simulated and measured threshold voltage degradation rates.

The Vth degradation rate of four kinds of devices was explored through experiments, and the bilayer dielectrics MIS-HEMT showed the lowest degradation rate, as seen in Figure 5, which is in accordance with the simulation results, strongly supporting the effectiveness of the simulation. The higher degradation in the other three devices may have been caused by the Ga-O bond and other dangling bonds at the interface induced during the manufacturing process, which may cause deep interface states [21]. These native defects may transfer into more defects after proton irradiation, leading to more severe degradation.

**Figure 6:**
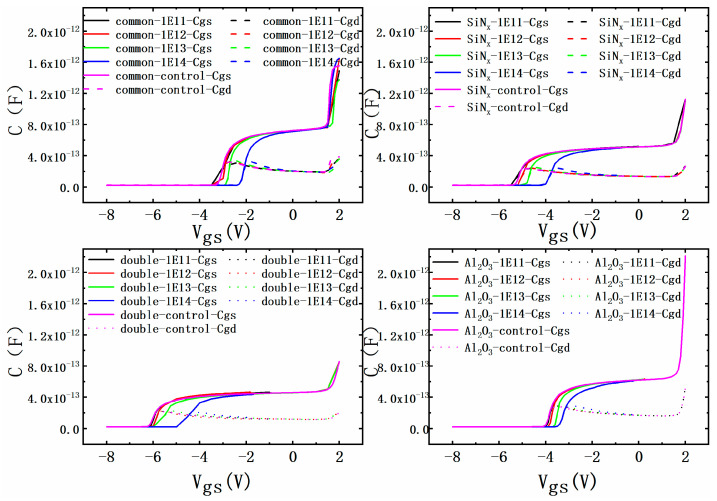
Capacitance degradation of simulation with irradiation fluence in different devices after proton irradiation.

The shift in C-V curves of Cgs and Cgd for different irradiation cases was also studied through simulations, and the results are shown in Figure 6. The C-V curves of all four kinds of devices changed slightly when the fluence was below 10^13^. When the fluence reached 10^13^, the C-V curves of the bilayer dielectrics MIS-HEMT still changed little, while the other three devices showed a clear shift, which means that the bilayer dielectrics MIS-HEMT takes effect at higher fluence, which is attributed to its stronger surface protection due to the higher displacement threshold energy of Al_2_O_3_. When the fluence grew to 10^14^, all devices showed a great shift, which may be attributed to the formation of trapped charges and greater lattice damages in the devices [22]. Similar results have also been reported [17]. The shift in the experimental Cgs results in four devices was 0.54 V, 0.41 V, 0.44 V, and 0.47 V, respectively, in the common Schottky gate HEMT, the single SiN_x_ MIS-HEMT, the bilayer dielectrics MIS-HEMT, and the single Al_2_O_3_ MIS-HEMT at the proton fluence of 10^14^. The larger shift in the single Al_2_O_3_ MIS-HEMT in the experimental results than in the simulation results may contribute to the defects introduced in the fabrication process.

**Figure 7:**
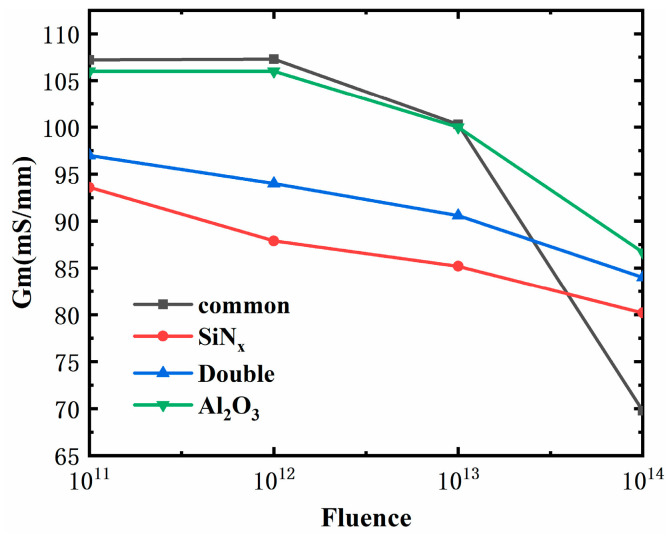
Simulation results of Gmmax degradation with irradiation fluence in different devices after proton irradiation.

The Gmmax degradation of four kinds of devices is explored in Figure 7. The Gm decreased largely in both the common Schottky gate HEMT and the MIS-HEMT with Al_2_O_3_, while the Gm decreased mildly in the MIS-HEMT with SiN_x_ and the MIS-HEMT with bilayers. The results of the Gm degradation rate (△Gm/Gm0) are shown in Table 5. The Gm degradation rates of the MIS-HEMT with SiN_x_ and the MIS-HEMT with bilayers were relatively smaller, at 9.7% and 12.28%w while the degradation rates in the other two devices were larger, especially in the common Schottky gate HEMT, reaching as high as 34.58%. This means that the gate control capability of the MIS-HEMT with SiN_x_ and the MIS-HEMT with bilayers degraded less than the other two devices. This difference in Gm degradation may be because SiN_x_ can passivate the traps in the device surface and interface. Fewer traps may generate fewer defects after proton irradiation in the device, causing smaller damage to the region under the gate, which can influence the gate control capability.

**Figure 8:**
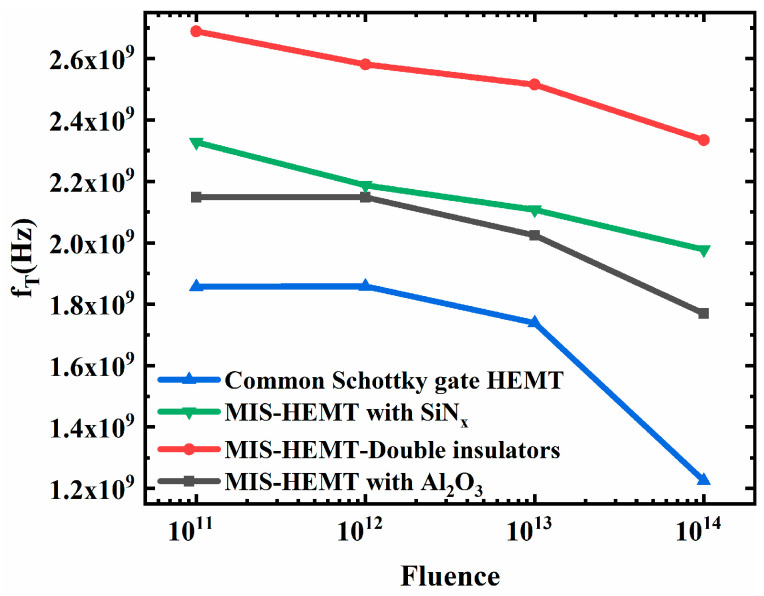
Simulation results of frequency degradation with irradiation fluence in different devices after proton irradiation.

What is more, the cutoff frequency (f_T_) at Vds = 6 V under different fluences was compared in four devices, as shown in Figure 8. The bilayer dielectrics MIS-HEMT ranked first at a different fluence, higher than that of the single-dielectric-layer MIS HEMT with a SiN_x_ layer and with an Al_2_O_3_ layer, whereas the lowest cutoff frequency occurred in the common Schottky gate HEMT. As the proton fluence increased, the cutoff frequencies of the four devices were reduced, suggesting that particle irradiation will deteriorate the frequency characteristics of the devices. Nevertheless, the reduction in f_T_ at different fluence in the bilayer dielectric MIS-HEMT was lower than the other devices. It has been reported that the high-frequency performance of GaN-based devices will be degraded when charge trapping occurs in these devices [15,23,24]. The cutoff frequency degradation rate (△f_T_/f_0_) of the four devices was also calculated with the simulation results and is listed in the Table 6. The smaller degradation in the bilayer dielectric MIS-HEMT suggests that the frequency characteristic of the bilayer dielectric MIS-HEMT was more stable when the proton fluence increased. Higher f_T_ and lower reduction in f_T_ at different fluence in MIS-HEMTs indicate the charge trapping has been suppressed [24], thereby improving the performance degradation of the device. The MIS-HEMT, especially with bilayer dielectrics, is therefore more suitable for microwave applications [24]. This agrees with the defects simulation of structural material. The fewer the defects, the smaller the degradation. The experimental results of the threshold voltages and C-V curves were consistent with the simulation results, suggesting that the bilayer dielectrics MIS-HEMT has better irradiation reliability.

It can be seen from the simulation and experimental results above that the bilayer dielectrics MIS-HEMT showed better radiation resilience. This may be related to the effect of the upper Al_2_O_3_ and the lower SiN_x_ layer. The higher displacement threshold energy of Al_2_O_3_ decreased defects induced by particle incidence, and the lower SiN_x_ layer provided better surface passivation. The combined effect resulted in better performance after irradiation.

## 4. Conclusions

This paper investigates the proton radiation effects on the bilayer dielectrics SiN_x_/Al_2_O_3_ MIS-HEMT, the common Schottky gate HEMT, and single dielectric layer MIS-HEMT with SiN_x_ and with Al_2_O_3_. Different proton particle fluences are adopted in our simulations and experiments, and the degradation of structural material and electrical performance of the common Schottky gate HEMT and three different kinds of MIS-HEMTs are explored. Simulation and experimental results of structural material degradation and electrical performance degradation reveal that the bilayer dielectrics MIS-HEMT performs best after irradiation and has better radiation resilience.

## Figures and Tables

**Table 1:** Basic electrical results (experimental) in different devices.

Different Devices	The Measured 2DEG Density ns (cm^−2^)	The Calculated Equivalent Mobility μ (cm^2^/Vs)
Common	8.41 × 10^12^	130.99
SiN_x_	6.02 × 10^12^	140.00
Bilayer	4.99 × 10^12^	251.61
Al_2_O_3_	6.88 × 10^12^	160.55

**Table 2:** SRIM simulation results in different structural materials.

Defects	SiN_x_	Al_2_O_3_	Bilayer
Total replacement	292/ion	285/ion	139/ion
Total vacancy	282.3/ion	295/ion	142/ion
Replacement collision	10/ion	10/ion	3/ion

**Table 3:** Experimental degradation results in different devices after the irradiation fluence of 10^14^.

Different Devices	Degradation of the Maximum On-Current
Common	78.5%
SiN_x_	18.8%
Bilayer	7.5%
Al_2_O_3_	38.0%

**Table 4:** Simulation results of degradation rate of Vth.

Fluence	Degradation Rate of Vth (%)			
	Common	SiN_x_	Bilayer	Al_2_O_3_
1 × 10^11^	16.3	11.4	6.7	11.0
1 × 10^12^	20.5	14.9	10.3	14.3
1 × 10^13^	25.5	19.8	15.5	19.5
1 × 10^14^	44.8	35.9	32.6	35.2

**Table 5:** Results of degradation of Gm after the irradiation fluence of 10^14^.

Different Devices	Degradation of Gmmax (Simulation)	Degradation of Gmmax (Experiment)
Common	34.58%	9.6%
SiN_x_	12.28%	6.0%
Bilayer	9.7%	5.6%
Al_2_O_3_	19.87%	6.3%

**Table 6:** Simulation results of degradation of cutoff frequency after the irradiation fluence of 10^14^.

Different Devices	Degradation of Cutoff Frequency
Common	33.7%
SiN_x_	12.4%
Bilayer	9.5%
Al_2_O_3_	18.9%

## Data Availability

The raw data supporting the conclusions of this article will be made available by the authors on request.
